# Factors Impacting Plant-Based Meat Product Consumption: A Consumer Survey Conducted in a New First-Tier City in China

**DOI:** 10.3390/foods13213496

**Published:** 2024-10-31

**Authors:** Cong Shen, Xinyao Wu, Enheng Zhang, Ying Liu

**Affiliations:** 1 School of Management, Henan University of Technology, Zhengzhou 450001, China; zzwgy12345@163.com; 2 School of International Education, Henan University of Technology, Zhengzhou 450001, China; 211170500522@stu.haut.edu.cn; 3 School of Artificial Intelligence and Big Data, Henan University of Technology, Zhengzhou 450001, China; z13598600075@163.com; 4 College of Food Science and Engineering, Henan University of Technology, Zhengzhou 450001, China

**Keywords:** plant-based meat, consuming behavior, structural equation modeling, bibliometric analysis

## Abstract

In recent years, the worldwide plant-based meat sector has undergone substantial and rapid expansion. The swift advancement of plant-based meat products in the Chinese market is ascribed to changes in customer dietary preferences. To accelerate the rapid expansion of China’s plant-based meat sector, it is essential to conduct research on consumer demand trends. Citespace was utilized in this study to conduct a bibliometric analysis of research pertaining to plant-based meat. A study model was then created to analyze the primary elements affecting the consumption behavior of plant-based meat products. This study employs Zhengzhou as a case study to construct a research model to examine consumers’ inclination to purchase plant-based meat products. The model is derived from survey data obtained from 570 consumers. The findings indicate that the characteristics of plant-based meat products significantly influence consumers’ purchase intentions and consumption behaviors by shaping their perceptual activity. Price rationality, technical security, and flavor richness are three principal factors influencing customer purchasing of plant-based meat products. The perceived value and trust of consumers can somewhat mediate the influence of plant-based meat consumption behavior. This study offers significant insights into purchasing intentions and consumer behavior in first-tier cities in China. The outcomes of this study can provide a beneficial framework for imitation meat producers to improve product development and stimulate customer interest in the plant-based meat market.

## 1. Introduction

In recent years, the global industry for plant-based meat has experienced fast expansion as an inventive substitute for regular meat. The reputable Euromonitor stated that the global market for plant-based meat would reach a value of over USD 30 billion in 2025. China’s plant-based meat sector is unquestionably a significant and unavoidable component of this worldwide trend. China has enormous development potential and a wide range of market opportunities as the world’s largest producer and consumer of meat products. A growing number of customers are starting to take notice of plant-based meat as a new and healthy food option as a result of rising meat demand and rising living standards in recent years. By 2025, China is predicted to hold more than half of the world market for plant-based meat [1].

Meat substitutes made from plants have special benefits. First off, there are clear benefits to plant-based meat in terms of lowering biological carbon emissions as compared to conventional animal meat products. Because plant-based meat does not need to be farmed and has a high energy conversion rate, it can help ease growing ecological stress and lower carbon emissions from agricultural land. According to related research, when compared to comparable animal meat products, plant-based meat products can more than quadruple carbon emissions reduction. Second, plant-based meat is healthier and more environmentally friendly than traditional animal meat products [2]. It has zero cholesterol, high levels of dietary fiber and protein, and other benefits that can help prevent health issues like fatty liver and hyperlipidemia. Additionally, it can consistently meet the increasing demands of consumers for a balanced diet. China’s market for plant-based meat has great potential for growth because of the country’s large population and the worldwide food shortages brought on by extreme weather. The Chinese government has been outlining the path for the growth of the new protein industry since 2023. It is stated that the Chinese government is willing to aggressively advance the biotechnology and biological industries, as well as plant, animal, and microorganism sources of protein [3].

China, one of the most populous nations worldwide, is a major contributor to the scarcity and security of food brought on by climate change. In this regard, aggressively growing the market for plant-based meat is crucial to reducing the strain on the world’s food supplies and is also a necessary step toward the food industry’s sustainable growth. Although the Chinese government actively promotes and encourages the growth of the plant-based meat sector, plant-based meat technology is still in its early stages of development. Consumers’ acceptance of plant-based meat may be impacted by the technological, flavor, and experience issues that plant-based meat products still face. Using the Chinese market as an example, while Starbucks and KFC continued to introduce new plant-based meat products in 2020 and Chinese consumers showed a great deal of interest in these products during the initial listing phase, the products’ ongoing sales did not meet expectations. This demonstrates that while plant-based meat substitutes in the Chinese market may spark customers’ enthusiasm in the near term, consumers’ purchase willingness and real consumption patterns still require additional investigation. Consequently, comprehensive studies on consumers’ acceptance and intention to purchase plant-based meat products are beneficial for improving consumer demand, optimizing product attributes, and offering strong theoretical backing and useful direction for the growth and success of the plant-based meat industry. Therefore, it is crucial to conduct in-depth market research on customers’ intentions to purchase plant-based meat products in order to understand consumer demand for these goods and support the industry’s healthy growth. While there have been some discussions among researchers, most of them have focused on relatively developed markets in Europe and America [4]. Studies on consumer acceptance of plant-based meat products in developing nations, particularly in China, a country with a high food demand, are scarce [5]. Thus, this study conducted a thorough analysis of the variables influencing consumers’ desire to purchase plant-meat products in the Chinese market, drawing on theories pertaining to consumer perception and the attributes of such items. The necessary consumer group data from China’s new first-tier cities was gathered to create the study model of plant-meat product consumption willingness in order to offer pertinent theoretical support for the market for plant-based meat’s rapid and healthy development.

## 2. Literature Review

As awareness of healthy diets and environmental conservation grows, plant-based meat products have garnered significant commercial attention as a novel meat substitute in recent years. The eating of plant-based meat satisfies consumer demands for a nutritious diet while aligning with environmental preservation principles, thus rendering the research on plant meat product consumption behavior both practically significant and theoretically valuable. The current market for plant-based meat products exhibits a diverse array of options. In addition to liking conventional offerings like plant-based meat pieces and sausages, consumers also like creative goods such as plant-based meat burgers and pizzas. The swift advancement of e-commerce platforms has enhanced consumer convenience in purchasing plant-based meat products [6]. The research of the consumption features of plant meat products in pertinent studies reveals that consumers’ purchasing decisions are influenced by various multidimensional aspects.

Firstly, some experts think that certain attributes of plant-based meat substitutes will influence customers’ propensity to purchase such substitutes. Research has indicated that consumer acceptance of plant-based meat substitutes will depend on a number of factors, including cost, flavor, natural material sourcing, safety of technology, and adequate nutrition [4,7,8,9,10,11]. In order to investigate the possible elements that influence consumer willingness, the aforementioned factors are mostly based on the product qualities of plant-based meat.

Secondly, certain researchers have focused on the behavioral traits of customers. The behavioral traits of customers also have an impact on their propensity to purchase plant-based products, as the researchers discovered. For instance, studies have shown that a variety of factors, including age, location, education level, perception of regulations, health cognition, perceived behavior, and knowledge level, influence consumers’ intentions to make a purchase [7,9,10,12,13,14,15,16,17,18,19].

Thirdly, from the standpoint of consumption values, recent trends in food consumption among consumers exhibit characteristics of sustainability, environmental protection, and low carbon emissions. An increasing number of customers are opting to diminish their consumption of conventional meat for health considerations, and plant-based meat alternatives may serve as a beneficial substitute for lower fat and cholesterol levels [20,21]. Simultaneously, the production process of plant-based meat exerts a reduced environmental impact compared with conventional meat, aligning with the increasing environmental consciousness of contemporary customers [22,23].

To systematically categorize and assess the pertinent research on vegetable meat market consumption, we then employed bibliometric methodologies to gather a substantial volume of current literature in connected domains. This study conducted a complete analysis and discussion of the material after first sorting and searching the relevant literature on the topic of plant-based meat alternatives using Citespace 6.3.R1 software. The data came from the Social Sciences Citation Index (SSCI) and Science Citation Index Expanded (SCI-E) databases under the Web of Science Core Collection. This database is the most comprehensive and extensively used scientific database across most areas. The information encompassed all published works on plant-based meat in these two databases between 2014 and 2024. The following is the retrieval technique. TS = [(“plant-based meat”, OR “artificial meat”, OR “cultivated meat”, OR “cultured meat”) AND (“consum*”)] were the research topic phrases. The English language article types included paper and review. A total of 557 items were looked up.

**Figure 1:**
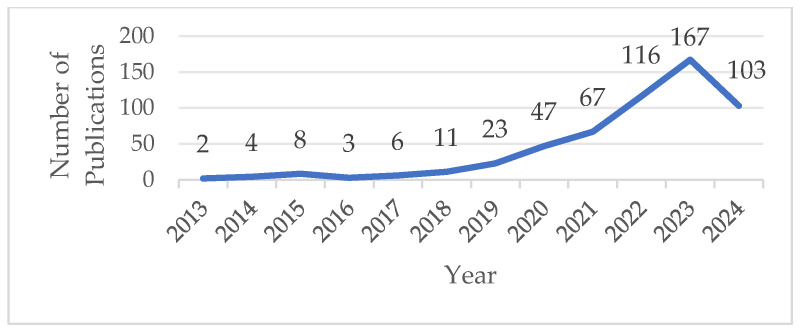
Publication output performance.

When viewed from a temporal distribution viewpoint, the number of research publications pertaining to plant-based meat substitutes demonstrated a general upward trend between 2013 and 2024. After 2019, related research expanded quickly. This could be because the world’s first plant-based meat company debuted on the NASDAQ in 2019, and the plant-based meat market has subsequently experienced explosive growth. The literature started to spread by 2022. The increased public emphasis on eating healthily may be connected to this. Because it has more protein and less fat than regular meat, plant-based meat is growing in popularity as an alternative. Figure 1 displays the number of relevant publications and their trend over time.

**Figure 2:**
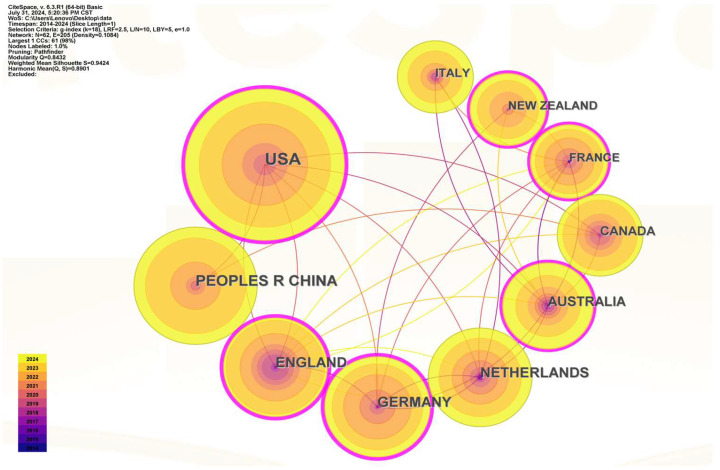
The cooperation network of the productive countries.

Figure 2 shows 205 lines and 62 nodes that represent the various countries’ research status. The thickness of the connecting lines shows the strength of the collaboration, while the size of the circle represents the quantity of papers published by each nation in the research field. Figure 2 illustrates that the majority of research on plant-based meat substitutes is conducted in wealthy nations in Europe and the US. The centrality parameter can be used to calculate the level of cooperation. The top three nations in terms of the intensity of international collaboration were Germany (0.22), the United States (0.37), and the United Kingdom (0.26). With 134 articles published, the United States placed first in the world. China and the United Kingdom came next, with 75 and 60 publications, respectively. The Netherlands and Germany were not far behind. Although Chinese plant-based meat has a sizable global market, it is evident from the published chart of the number of nations that there is still a dearth of pertinent consumer market research on the subject. Studies on the market for plant-based meat in China are essentially on par with those conducted in the UK, Germany, the Netherlands, France, Australia, New Zealand, North America, and Canada. The amount of research on the plant-based meat markets in China and the US still differs significantly.

**Figure 3:**
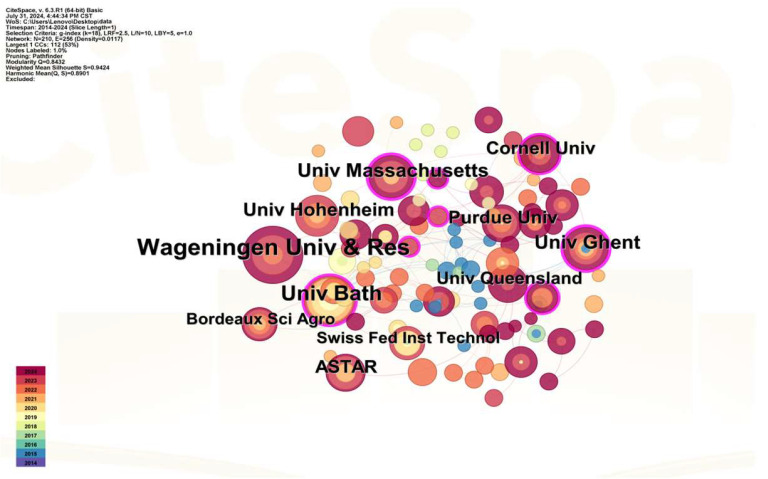
The cooperation network of institutions.

The network of institutional collaborations in research fields relevant to plant-based meat is depicted in Figure 3. The top three most prolific scientific institutions among them are Wageningen University & Research in the Netherlands (24 publications), the University of Bath in the UK (18 publications), and Ghent University in Belgium (14 publications). The collaboration network of research institutions in Figure 3 reveals that studies on plant-based meat remain in the nascent phase of exploration, as evidenced by Wageningen University & Research, which, despite having the highest publication count, has only 24 relevant studies in this domain. The analysis of the pertinent cooperation network metrics (N = 210, E = 256, density = 0.0117) indicates that the interconnections among diverse research institutes require further enhancement.

Table 1 demonstrates that the five journals with the greatest number of publications in plant-meat-related fields are *Trends in Food Science and Technology* (400), *Appetite* (378), *Meat Science* (364), *Food Quality and Preference* (323), and *Foods* (297). These journals hold significant positions in the field. The authors who are most frequently mentioned in this field are BRYANT C (262) and SIEGRIST M (206). This suggests that these authors have made significant contributions to related fields of research.

**Figure 4:**
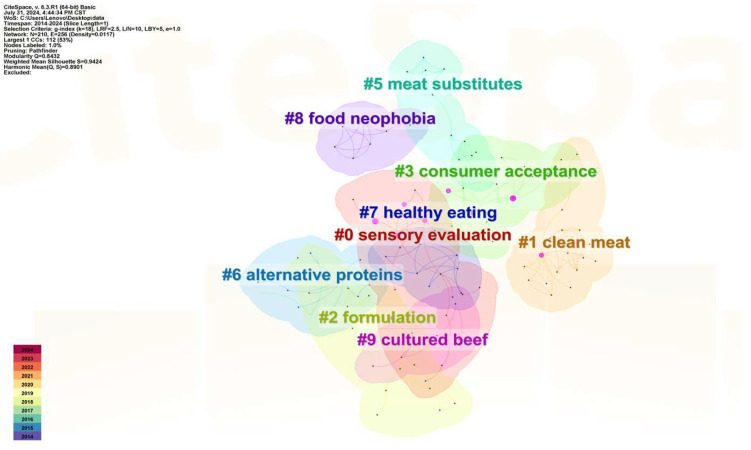
Literature cluster analysis.

The cluster analysis of linked studies indicates that the most active areas of study were compiled. The node type selected was Cited Reference. The term or document that had the highest frequency each year, accounting for 50% of the total, was chosen as one of the Top N and referred to as the Top 50. Simultaneously, the time interval was adjusted to 1 year for each segment. The data contain the names of the main groupings, which include clean meat, formulation, consumer acceptability, meat alternatives, alternative proteins, healthy eating, and food neophobia. The findings are presented in Figure 4. The graph’s goodness of fit was satisfactory, as evidenced by the data presented in Figure 4 (N = 210, E = 256, Density = 0.0117, Modularity Q = 0.8432 (>0.3), Mean Silhouette = 0.9424 (>0.4)). The Modularity Q measure determined whether the graph could be divided into clusters, whereas the Mean Silhouette measure determined the strength of the connections between documents inside each cluster. It is not difficult to determine from the cluster analysis that customer acceptance is a crucial area for further study in the market for plant-based meat (Cluster #3). Researchers are also focusing on some plant meat production technologies and production safety factors because plant meat products are emerging foods that differ from traditional meat foods (cluster #0, 1, 2, 6, 8).

**Figure 5:**
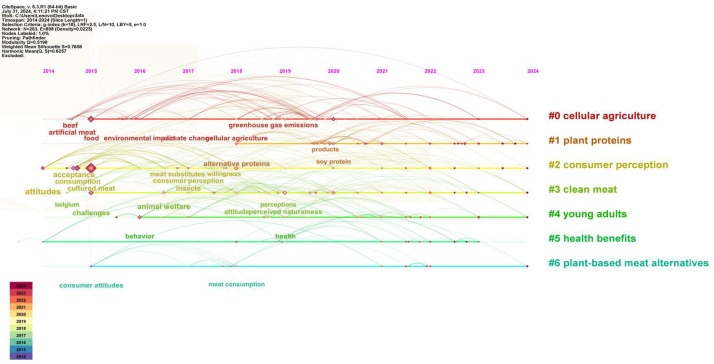
Keyword timeline analysis.

**Figure 6:**
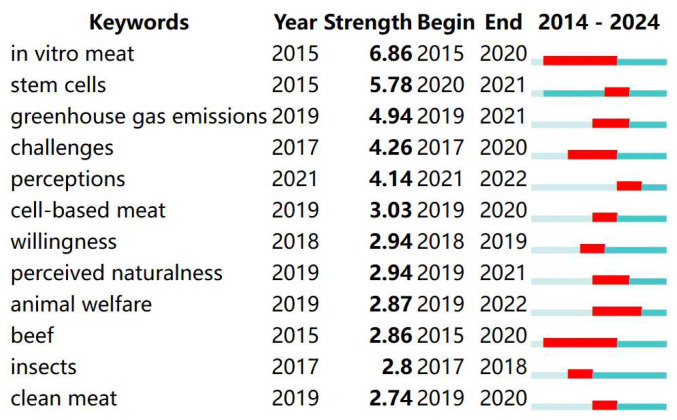
Top 12 keywords with the strongest citation bursts.

A time graph, which is constructed on the basis of keywords and their burst values, can effectively illustrate the research development pattern of the literature. To specify time parameters in CiteSpace, the node type is chosen, the TOP N value is set to 50, and the time slice is set to 1. Figure 5 displays the time graph for the keyword. Simultaneously, the study revealed that 10 out of the 12 most significant terms originated after the year 2020. Research on plant-based meat products has gained significant attention in recent years. Research trends in plant-based meat products encompass the exploration of technology, problems, and studies connected to consumption (refer to Figure 6).

The current analysis reveals that the studies that have already been conducted have either been based on the analysis of consumer behavior features or on the discussion of the properties of plant-based meat products. In actuality, the willingness to consume or consumption behavior may be influenced by both of these elements. Only a small number of studies do not fully account for multidimensional aspects. Furthermore, the majority of research on the consumption patterns of plant-based meat substitutes has concentrated on economically developed regions like Europe and North America [7,17,18]. By contrast, the consumption intentions of large developing nations like China, which consume a lot of food, are not as high as expected when it comes to the market for plant-based meat products. Thus, building on earlier research, this study gathers additional data and examines the main variables influencing Chinese consumers’ consumption patterns by developing a consumer behavior research model that incorporates the features of plant-based meat substitutes and consumer perception traits. By constructing the model and utilizing empirical test survey sample data, it offers some important guidelines for the rapid and healthy growth of China’s market for plant-based meat consumption.

## 3. Model Construction and Research Hypothesis

The term customer perceived value describes how executives assess the worth of goods or services that businesses offer. Zeithaml [27] introduced perceived value theory from the viewpoint of the consumer. Zeithaml thought that consumers would weigh the relative benefits and perceived costs of goods and services when making judgments about what to buy. In marketing [27], customer behavior [28], and service management [29], the notion of perceived value is frequently applied. This study examined the elements that influence customers’ purchasing behavior from the viewpoints of perceived qualities of plant-based meat products and consumers’ perceptions of those same characteristics according to perceived value theory.

The objective of this study was to construct a general research model of the desire to consume plant-based meat products by considering the attributes of plant-based meat products and the characteristics of consumer perception. We introduce the research hypothesis in the model, which posits that the characteristics of plant-based meat alternatives impact customers’ appraisals of those characteristics, subsequently affecting consumers’ intent to purchase and their patterns of consumption. Therefore, considering the perceived value and perceived trust of consumers, we propose the following hypothesis.

Technical security pertains to the utilization of plant protein and other raw materials transformed into meat analogs, ensuring that their production, consumption, and environmental impact adhere to safety standards. The confidence of customers in plant-based meat products is closely linked to their technical security [30]. The confidence that consumers have in plant meat products will be negatively impacted if there are any safety risks associated with the production process, such as pesticide residues, overuse of fertilizers, microbiological contamination, etc. Customers may decide not to purchase a product if they have concerns about its safety and instead hunt for other, safer food options. Furthermore, customers may consider technological safety to be a crucial aspect when assessing the worth of plant-based meat substitutes [31]. A product’s perceived value may increase if it has a high level of technical security because consumers will view it as more dependable and trustworthy. In light of this, this research puts forth the following hypotheses:

**H1.**  *Technical security has a positive and significant impact on consumers’ perceived value.*

**H2.**  *Technical security has a positive and significant impact on consumers’ perceived trust.*

Pricing sensitivity is a common trait among consumers purchasing plant-based meat products. When making a purchase, consumers must consider the cost-performance of plant-based meat products because they are typically more expensive than conventional meat [32]. Pricey products can give consumers the impression that they are better-quality or have a better flavor, but they can also give them the impression that they are overpriced or that there are more affordable options available. Customers may begin to question the genuineness of the product’s quality if it is priced too low. Customers will perceive more trust or value in the purchasing process if plant-based meat substitutes are reasonably priced [24]. In light of this, this research puts forth the following hypotheses:

**H3.**  *Price rationality has a positive and significant impact on consumers’ perceived value.*

**H4.**  *Price rationality has a positive and significant impact on consumers’ perceived trust.*

Flavor richness pertains to the specialized processing that enables plant-based meat products to replicate the taste and texture attributes of genuine meat, including juiciness, tenderness, and chewiness. The demands of consumers for food are becoming more varied and emphasize flavor and taste in addition to nutrition and health. Plant-based meat products offer a wide range of taste options, so they may satisfy the needs and taste preferences of diverse consumers. For instance, some customers like their meat to be soft, while others like it to be chewy. By modifying the recipe and production method, plant meat products can offer a range of flavor alternatives to satisfy a wide range of consumer preferences [33,34]. Consumers are more likely to accept and trust plant-based meat substitutes if they taste similar to the flavor, texture, and chewiness of real meat. In light of this, this research puts forth the following hypotheses:

**H5.**  *Flavor richness has a positive and significant impact on consumers’ perceived value.*

**H6.**  *Flavor richness has a positive and significant impact on consumers’ perceived trust.*

According to the current research, we discovered that depending on the circumstance, consumers’ buying intention is influenced differently by perceived value and perceived trust. First of all, after balancing the perceived benefits of a product or service against the cost of obtaining it, consumers’ perceived value is the overall assessment of the utility of that good or service [35]. After being first introduced to the topic of consumer buying by [36], perceived trust was shown by later researchers to directly affect customers’ propensity to purchase food [23]. More research is needed to determine how these perceived traits of customers may impact their purchasing intention and behavior in the artificial meat market. Consequently, the research hypotheses about the influence of consumers’ perceptual activity on purchase intention and consuming behavior are as follows.

**H7.**  *Perceived value has a positive and significant impact on consumers’ purchase intention.*

**H8.**  *Perceived trust has a positive and significant impact on consumers’ purchase intention.*

**H9.**  *Consumers’ purchase intention has a positive and significant impact on consuming behavior.*

**Figure 7:**
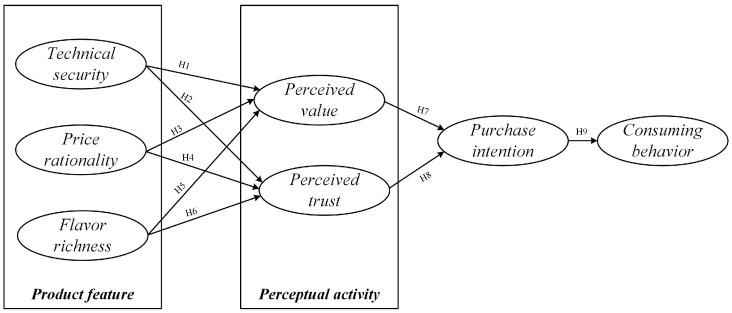
Research model of plant-based meat consuming behavior.

Figure 7 shows the research model diagram and corresponding hypotheses of factors affecting the consuming behavior of plant-based meat products.

## 4. Research Design

This research empirically analyzed and tested the key factors influencing consumers’ purchasing of plant-based meat products by building a research model of consumers’ purchasing behavior and using Zhengzhou, a typical important food city in central China, as an example. The questionnaire was mainly carried out and collected using the Chinese online questionnaire survey platform “WJX”. The platform has the advantages of convenient use, high data collection efficiency, data information security, and reliability. Structural equation modeling was appropriate for this study because it enabled us to address the relationship between several latent and explicit variables simultaneously, to investigate the internal relationship and path effect among variables in depth by building intricate causal relationship models, to address non-observed variables and take measurement errors into account, and to produce more thorough and accurate analysis results.

The following factors led to Zhengzhou, a well-known food town in central China, being chosen as the research site. First, Zhengzhou, the capital of a province in central China, is a significant food town with a sizable food market and commercial district. Second, Zhengzhou, one of China’s 15 new first-tier cities, has a GDP of RMB 1.36 trillion, ranking among the highest in the nation, and a population of 13 million, making it a prime location for plant-based meat products. Third, Zhengzhou’s market for plant-based meat substitutes has grown quickly at the same time as urban dwellers are more open to new ideas and have higher levels of knowledge, making this an ideal location for research. A total of 570 valid questionnaires were distributed via the online survey in this study, and 92.8% of the questionnaires were successfully recovered. The following demographic data relates to consumers.

In this study, a questionnaire survey was employed to confirm the model’s validity. The following three components make up the majority of the questionnaire design. In this investigation, a screening item was first established. We chose to interview customers who had bought goods made from plant-based meat. Second, this study used the five-point Likert scale approach to create pertinent problems in order to measure the variables. There were five possible values from 1 to 5, ranging from “strongly disagree” to “strongly agree”. Furthermore, we create pertinent inquiries for demographic factors. The mature magnitude of prior, pertinent investigations served as the primary inspiration for the question design in this investigation. Pre-research was conducted, and specialists in relevant domains evaluated the measured items. Table 2 displays the questionnaire’s descriptive statistical results. Constructs and survey questions are shown in Table 3.

The study was conducted in accordance with the Declaration of Helsinki, and ap-proved by the Ethics Committee of School of Management, Henan University of Technology (20240925).

## 5. Data Analysis and Hypothesis Testing

Using confirmatory factor analysis, the research assessed whether the variables developed in the previous investigations were sufficiently evident in this questionnaire. In this study, reliability, validity, and hypothesis testing analyses were conducted using SPSS and AMOS.

### 5.1. Reliability and Validity Analysis

The scale’s validity and reliability were examined using confirmatory factor analysis. As shown in Table 3, the current study used factor loading (STD) to assess each measurement item’s reliability and CR value to assess the model’s component reliability. The measured items’ factor load (STD) values were all more than 0.7, and the CR values all approached the 0.8 threshold, as per the acquired data. Every indication satisfied the requirements, showing that the scale’s stability and the model’s dependability are both strong [37]. Table 3 displays the reliability indicators’ test findings. Convergent validity tests and discriminant validity tests were the two categories under which the questionnaire validity test fell. Using Average Variance Extracted (AVE), the convergent validity was tested. Values larger than 0.5 suggest that the convergent validity is optimal, while values between 0.36 and 0.5 suggest that the convergent validity is adequate [38]. It was calculated that the model’s AVE values satisfied the necessary conditions, proving the scale’s good convergent validity. It is necessary to compare the dimension’s correlation coefficient matrix in order to perform a discriminant validity test. The arithmetic square root of AVE is shown in Table 4 as the diagonal bold type. The good discriminant validity between facets is indicated by the diagonal AVE root values, which are all greater than the Pearson correlation coefficient between this facet and other facets. The aforementioned information demonstrates that the model’s validity test results are good (as shown in Table 4).

Table 5 shows the goodness of fit test of the model. It can be seen that all indicators of the model are within the range required by the goodness of fit index. This shows that the overall level of the research model constructed by collected data is suitable.

### 5.2. Path Analysis

Firstly, the effects of plant-based meat product characteristics on consumers’ perceptual activity were analyzed. It can be seen from the results of hypothesis testing that the impact of plant-based meat product characteristics on consumers’ perceptual activity is significant.

Among them, technical safety, price rationality, and taste richness all have positive and significant effects on consumers’ perceived value (β = 0.232, *p* < 0.001; β = 0.297, *p* < 0.001; β = 0.225, *p* < 0.001). The results show that the characteristics of plant-based meat products can significantly affect the perceived value characteristics of consumers. Among them, price rationality is the most important factor affecting consumers’ perceived value, followed by technical security and flavor richness. Technical security, price rationality, and flavor richness all have positive and significant effects on consumers’ perceived trust (β = 0.212, *p* < 0.001; β = 0.136, *p* < 0.01; β = 0.249, *p* < 0.001). Among them, the influence of flavor richness and technical security on consumers’ perceived trust is higher, and the influence of price rationality is second. From the analysis results, it was found that the differentiation degree of the impact of plant-based meat product characteristics on consumers’ perceived value was not high, but there was a certain differentiation degree of product characteristics on consumers’ perceived trust. The reason behind this may be because at the level of trust, consumers are mainly concerned about the particularity of plant-based meat products that are different from traditional meat foods. Because the price of plant-based meat products in the market is relatively stable, and the price factor will become more competitive with the expansion of the market scale, so in comparison, in the level of trust, consumers pay more attention to the taste, flavor, and technical security of plant-based meat products.

Secondly, it analyzed the influence of consumers’ perceived characteristics on their purchase intention and consuming behavior. Among them, perceived value has a positive and significant impact on consumers’ purchase intention (β = 0.317, *p* < 0.001). Perceived trust has a positive and significant impact on consumers’ purchase intention (β = 0.392, *p* < 0.001). The influence of purchase intention on consuming behavior is also positive and significant (β = 0.105, *p* < 0.01). This is consistent with the findings of existing research on consumer trust and consumer perceived value. Only when consumers have trust in the product and think that the product has value will participation produce purchase intention, and then consumer behavior occurs.

The aforementioned conclusions provide insights for firms engaged in plant-based meat production. Primarily, as customers increasingly prioritize the logic of product pricing, a critical objective for plant-based meat manufacturers is to efficiently manage production costs while striving for market penetration and sustainable expansion. The rationalization and integration of supply chains, along with the promotion of large-scale production, can further diminish costs and enhance the market competitiveness of plant-based meat brands. Furthermore, with cost management, enhancing product quality is essential for plant-based meat makers to succeed in the market. For instance, producers of plant-based meat can innovate in purification and seasoning technologies to eliminate unpleasant tastes, such as the odor of beans, while replicating the taste and flavor of conventional meat to satisfy consumer preferences. Simultaneously, plant meat production firms must rigorously adhere to industry standards, guarantee the absence of prohibited additives in their products, enhance food safety management, and cultivate a robust and secure brand image, which is fundamental for earning consumer trust.

### 5.3. Intermediate Effect Test

It can be seen from the following table that the total effect path coefficient of technological security → consumer behavior is 0.394 (*p* < 0.001). The direct effect is 0.333 (*p* < 0.001). The path coefficient of technical security → perceived trust → purchase intention → consuming behavior is 0.009 (*p* < 0.05). This shows that technological security positively affects consumer behavior through perceived trust and purchase intention. The predicted path coefficient for the relationship between technical security, perceived value, purchase intention, and consuming behavior is 0.008 (*p* < 0.05). This indicates that technical security has a beneficial impact on consuming behavior through the mediating factors of perceived value and purchase intention. Therefore, perceived trust, perceived value, and purchase intention play a partial mediating role in the influence of technical security on consuming behavior.

The total effect path coefficient of price rationality → consuming behavior is 0.284 (*p* < 0.001). The direct effect is 0.236 (*p* < 0.001). The path coefficient of price rationality → perceived trust → purchase intention → consuming behavior is 0.006 (*p* < 0.05); this shows that price rationality positively affects consuming behavior through perceived trust and purchase intention. The path coefficient of price rationality → perceived value → purchase intention → consuming behavior is 0.010 (*p* < 0.05); this shows that price rationality positively affects consuming behavior through perceived value and purchase intention. Therefore, perceived trust, perceived value, and purchase intention play a partial mediating role in the influence of price rationality on consuming behavior.

The total effect path coefficient of flavor richness → consuming behavior is 0.205 (*p* < 0.001). The direct effect is 0.132 (*p* < 0.05). The path coefficient of flavor richness → perceived trust → purchase intention → consuming behavior is 0.012 (*p* < 0.05). This shows that flavor richness positively affects consuming behavior through perceived trust and purchase intention. The path coefficient of flavor richness → perceived value → purchase intention → consuming behavior is 0.009 (*p* < 0.05). This shows that flavor richness positively affects consuming behavior through perceived value and purchase intention. Therefore, perceived trust, perceived value, and purchase intention play a partial mediating role in the influence of flavor richness on consuming behavior.

**Figure 8:**
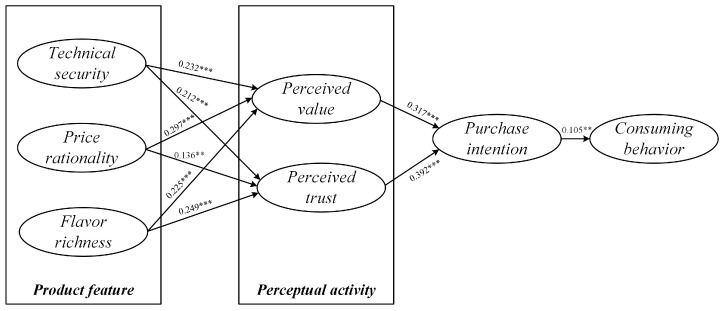
Path analysis of model hypothesis testing, ** indicates *p* < 0.01, *** indicates *p* < 0.001.

The model diagram and the results of the model hypothesis testing are shown in Figure 8 and Table 6, respectively.

## 6. Conclusions

This research examined the elements that impact the consuming behavior of 570 customers of plant-based meat products in a new first-tier city in China. It revealed that perceived value and perceived trust are the primary factors that directly influence consumers’ propensity to purchase plant-based meat products. Variables such as price rationality, technical security, and flavor richness have an indirect impact on customers’ purchase intention and consuming behavior by influencing their perception of these attributes.

The findings of this study offer valuable insights for plant-based meat manufacturers and sales organizations. Plant-based meat producers can minimize product expenses by establishing factories in China and innovating cost-effective alternative technologies. This approach helps alleviate the financial burden associated with raw materials and labor in the local production of plant-based meat while also facilitating the widespread manufacturing of plant-based meat products. Furthermore, the development of more sophisticated purification technologies can be pursued to eliminate the bean aroma in plant-based meat products and enhance their taste and quality. Furthermore, it is imperative for companies to adhere rigorously to industry standards during the production of plant-based meat products. They should enhance market self-regulation, refrain from unauthorized use of additives, maintain a reputable health brand, and cultivate consumer confidence. To enhance consumer inclination toward purchasing plant-based meat products and ensure sustained product popularity, the retail sector can minimize product prices within the sales chain, thereby aligning the cost of artificial meat foods with that of conventional meat. Retailers have the ability to enhance consumers’ experience by utilizing marketing, preferential treatment, tasting, and other methods. This can boost consumers’ perceptual activity, thereby increasing their propensity to purchase plant-based meat products.

The study’s online survey sampling method may have certain restrictions. For instance, Zhengzhou, a representative of China’s new first-tier metropolises, was the primary subject of our study. Zhengzhou has the drawback of being a relatively single-city pick despite being a representative city. Consumers in additional cities may be the subject of future research.

## Figures and Tables

**Table 1:** Citation analyses of journals and authors.

Ranking	Cited Journal	Counts	Ranking	Cited Authors	Counts
1	*Trends in Food Science and Technology*	400	1	BRYANT C [10]	262
2	*Appetite*	378	2	SIEGRIST M [24]	206
3	*Meat Science*	364	3	VERBEKE W [25]	189
4	*Food Quality and Preference*	323	4	POST MJ [26]	181
5	*Foods*	297	5	WILKS M [7]	160

**Table 2:** Description of sample characteristic distribution.

Demographic Variables	Items	Frequency	Percentage
Gender	Male	325	57.02%
	Female	245	42.98%
Age	20 and under	51	8.95%
	21–30 years old	131	22.98%
	31–40 years old	152	26.67%
	41–50 years old	124	21.75%
	51–60 years old	70	12.28%
	Over 60	42	7.37%
Education background	High school education and below	143	25.09%
	Undergraduate degree	167	29.30%
	Master’s degree or above	260	45.61%
Monthly household income	RMB 5000 and below	188	32.98%
	RMB 5001–10,000	302	52.98%
	RMB 10,001 and above	80	14.04%

**Table 3:** Constructs and reliability test.

Variable	Measurement Item	STD	CR	AVE	Reference
Technical security	Plant-based meat is produced without the use of any harmful ingredients.	0.803	0.914	0.781	[9]
	Production technology is comparatively advanced and it is safe to consume.	0.971			
	Plant-based meat products can reduce the insecurity of traditional meat products.	0.869			
Price rationality	Plant-based meat products in the market are reasonably priced.	0.798	0.901	0.753	[7]
	Plant-based meat products have price advantages compared with meat products.	0.965			
	The cost of plant-based meat products will continue to decrease as production scales up.	0.832			
Flavor richness	Compared with traditional meat products, plant-based meat products have a unique taste and flavor.	0.839	0.902	0.755	[4]
	Plant-based meat has a rational formula to enhance the flavor of the product.	0.904			
	Plant-based meat maintains the natural flavor while tasting quite similar to regular meat.	0.862			
Perceived trust	I think the promotion about plant-based meat products in the market is real.	0.838	0.881	0.711	[7]
	I think plant-based meat products in the market are healthy.	0.857			
	I think the plant-based meat products in the market are safe to eat.	0.835			
Perceived value	Purchasing plant-based meat products has helped me become more knowledgeable.	0.837	0.891	0.732	[10]
	Plant-based meat products increase my food choices.	0.889			
	I’ve been able to eat healthier foods since the development of plant-based meat alternatives.	0.839			
Purchase intention	I’m willing to take the initiative to try plant-based products.	0.878	0.896	0.742	[18]
	I will add plant-based meat products to my consumption plan.	0.862			
	If there’s a new plant-based meat product, I’d like to try it.	0.843			
Consuming behavior	I will continue to buy plant-based meat products for some time to come.	0.805	0.869	0.688	[10]
	I will increase the frequency of repeated purchases of plant-based meat products.	0.840			
	I think the consumption of plant-based meat products is a good experience.	0.843			

**Table 4:** Discriminant validity test.

	Discriminant Validity (Pearson Correlation)						
	1	2	3	4	5	6	7
1 Technical security	0.884						
2 Price rationality	0.528	0.868					
3 Flavor richness	0.491	0.484	0.869				
4 Perceived trust	0.491	0.456	0.513	0.843			
5 Perceived value	0.483	0.507	0.477	0.499	0.856		
6 Purchase intention	0.534	0.523	0.501	0.51	0.477	0.861	
7 Consuming behavior	0.655	0.605	0.545	0.555	0.452	0.559	0.829

**Table 5:** Index of model goodness of fit.

Model Fitting	CMIN/DF	NFI	IFI	TLI	CFI	RMSEA
Criterion	less than 3.00	greater than 0.90	greater than 0.90	greater than 0.90	greater than 0.90	less than 0.08
Observed value	2.329	0.955	0.974	0.968	0.974	0.048

**Table 6:** Hypothesis test result.

Hypothesis	Estimate	S.E.	C.R.	Result
Technical security → Perceived value	0.232	0.049	4.771	H1 established
Technical security → Perceived trust	0.297	0.052	5.713	H2 established
Price rationality → Perceived value	0.225	0.046	4.923	H3 established
Price rationality → Perceived trust	0.212	0.049	4.287	H4 established
Flavor richness →Perceived value	0.136	0.053	2.575	H5 established
Flavor richness → Perceived trust	0.249	0.047	5.286	H6 established
Perceived value → Purchase intention	0.317	0.050	6.349	H7 established
Perceived trust → Purchase intention	0.392	0.051	7.698	H8 established
Purchase intention → Consuming behavior	0.105	0.038	2.744	H9 established

## Data Availability

The original contributions presented in the study are included in the article, further inquiries can be directed to the first author.
